# Recent Advances in Preventing Adverse Reactions to Transfusion

**DOI:** 10.12688/f1000research.7048.1

**Published:** 2015-12-17

**Authors:** Thomas S Rogers, Mark K Fung, Sarah K Harm

**Affiliations:** 1 Blood Bank & Transfusion Medicine, University of Vermont Medical Center, Burlington, Vermont, 05401, USA; 2 Department of Pathology and Laboratory Medicine, University of Vermont College of Medicine, Burlington, Vermont, 05401, USA

**Keywords:** Blood Transfusion, Transfusion Related Acute Lung Injury, TRALI, Transfusion Associated Circulatory Overload, TACO, Human leukocyte antigens, Human leukocyte antigen antibodies

## Abstract

The spectrum of adverse reactions to blood product transfusion ranges from a benign clinical course to serious morbidity and mortality.  There have been many advances in technologies and transfusion strategies to decrease the risk of adverse reactions. Our aim is to address a few of the advancements in increasing the safety of the blood supply, specifically pathogen reduction technologies, bacterial contamination risk reduction, and transfusion associated acute lung injury risk mitigation strategies.

## Introduction

The transfusion of blood products is never without risk. Complications such as transfusion-transmitted infectious diseases (TTIDs), antibody formation to red and white blood cells, preformed cytokines, and sudden increases in intravascular volume, to name a few, may result in severe health consequences to the transfusion recipient. A large focus of the transfusion medicine community has been to decrease the risk of TTIDs such as human immunodeficiency virus (HIV), hepatitis B, and hepatitis C (see Table 1) through extensive donor infectious disease testing, removal of monetary compensation to blood donors, and enhancement of the donor health history questionnaire ^1– 4^. Despite the successes in reducing the risk of TTIDs, there remain the risks of sepsis due to bacterial contamination, transmission of unknown pathogens, and numerous non-infectious complications, some of which have emerged as leading causes of fatalities due to blood product transfusion.

**Table 1.** Transfusion-transmitted viruses. Current testing of blood donors miss these viruses if the donors are in the “window period” when they are infected but do not yet test positive ^1– 4^.

Transfusion-transmitted viruses	Risk per unit transfused
Human immunodeficiency virus	1:1,000,000 to 1:5,000,000
Hepatitis C virus	1:1,100,000 to 1:10,000,000
Hepatitis B virus	1:400,000 to 1:1,200,000

In 2014, transfusion-related acute lung injury (TRALI) was the leading cause of death due to blood product transfusion in the United States, followed by transfusion-associated circulatory overload (TACO) ^5^. Since 2010, 41% of transfusion-related fatalities were due to TRALI, 22% due to TACO, and microbial infection accounted for 8% (see Table 2). Unfortunately, there are no laboratory tests to prevent TACO in transfusion recipients. Thus, transfusion services are left with tools such as physician and patient education to recognize clinical signs and symptoms of TACO and help clinicians identify which patients might be most sensitive to sudden increases in intravascular volume. On the other hand, there are newer testing-based strategies to help prevent both TRALI and microbial infection, which once universally incorporated should further reduce the risk of these complications of transfusion.

**Table 2.** Transfusion fatalities reported to the Food and Drug Administration in the United States from fiscal year (FY) 2010 to FY2014 ^5^.

Causes	No. Cases FY2010–FY2014	%
TRALI and possible TRALI	72	41
TACO	38	22
Hemolytic transfusion reaction (non-ABO)	25	14
Microbial infection	15	8
Hemolytic transfusion reaction (ABO)	13	7
Anaphylaxis	10	6
Other*	3	2
TOTAL	176	100

Other: FY2010 and FY2011: Graft vs. host disease

FY2014: Hypotensive reaction

Additional non-infectious complications of transfusion, while not the leading causes of death, pose serious risks to recipients of blood products. Immunomodulation, nosocomial infection, and other consequences of biologic response modifiers (i.e. byproducts of the red blood cell and platelet storage lesion) may lead to transfusion-related morbidity and mortality ^6– 8^. Mitigation of these risks may include leukoreduction, byproduct removal by saline washing blood products, and/or using a restrictive transfusion strategy ^9– 11^. A recent meta-analysis showed that a restrictive transfusion strategy in patients with critical illness or bleeding, using a hemoglobin transfusion trigger of <7 g/dL, resulted in a significant reduction in cardiac events, rebleeding, bacterial infections, and total mortality when compared to a less restrictive (more liberal) strategy ^12^. However, it is well known that certain patient populations (e.g. acute coronary syndrome) may require higher hemoglobin transfusion triggers ^13^. In addition, recent randomized controlled clinical trials in critical care and cardiac surgery patients showed no difference in mortality when receiving fresh versus older red blood cell units ^14, 15^. Therefore, judicious use of blood products and avoidance of unnecessary transfusion in combination with leukoreduction (and saline washing when clinically indicated) provides the best defense against many of the non-infectious complications of transfusion.

Despite the best efforts of the transfusion medicine community, infectious and non-infectious risks of transfusion remain a problem for transfusion recipients. Ongoing studies continue to discover the consequences of blood product storage, the impact biologic response modifiers have on patient outcomes, the optimal triggers for transfusion, and the detection of pathogens in the blood supply. As we cannot address each and every improvement the transfusion medicine community has made to make the blood supply as safe as possible, we will briefly describe some of the newer strategies adopted by blood centers and hospital transfusion services to help prevent adverse reactions to blood product transfusions. Specifically, we will discuss general pathogen reduction (PR) technologies, improvements that increase the sensitivity of screening for bacterial contamination in platelet products, and the newest TRALI risk mitigation strategies for plasma and platelet products.

## Pathogen reduction technologies

The safety of blood product transfusion has increased greatly due to an extensive donor health history questionnaire and sophisticated donor infectious disease testing, yet the risk of pathogen-related complications in blood product recipients remains. PR technologies include using solvent and detergent, a psoralen compound, or riboflavin, the latter two combined with ultraviolet light, to render pathogens non-infectious (see Table 3) ^16^. The goal of PR is zero risk from existing and emerging pathogens in blood products. New technologies reduce but unfortunately do not eliminate the risk of viruses and microbial infection, mentioned in both Table 1 and Table 2. For example, certain *Klebsiella pneumonia* and *Bacillus cereus* microbes are not 100% killed by PR ^17, 18^. PR technologies have been slow to market in the United States as opposed to Europe, where PR has been in use for over 10 years (see Table 3) ^16, 19, 20^. While PR technology for platelets and plasma is in its infancy in the United States, the European experience has been positive, with only two transfusion-transmitted infections out of 1681 transfusion-related incidents reported in 2014 ^21^. In addition, a report on over 50,000 PR plasma transfusions showed no significant difference in adverse events (mostly allergic in nature) compared to non-PR plasma ^22^. PR for red blood cells is still in United States phase 2 and European phase 3 studies. Potential benefits of PR red blood cells include reducing the risk of current transfusion-transmitted infections to essentially zero, albeit with risks including alloimmunization and increased cost; however, the true rate of transfusion-transmitted infections has yet to be determined ^4^.

**Table 3.** Pathogen Reduction technology development and approval ^16, 19, 20^.

Component	Company	Technique	Status
Plasma	Cerus Corporation INTERCEPT™	Amotosalen + UVA light	- CE marked 2006 - FDA approved 2014
	Terumo BCT Mirasol ^®^	Riboflavin + UV light	- CE marked 2008
	Macopharma Theraflex ^®^	UVC (254nm) light	- CE marked 2009
	Octapharma OctaPlas ^®^	Solvent detergent	- CE marked 2009 - FDA approved 2013
Platelets	Cerus Corporation INTERCEPT™	Amotosalen + UVA light	- CE marked 2002 - FDA approved 2014
	Terumo BCT Mirasol ^®^	Riboflavin + UV light	- CE marked 2007 - U.S. phase III clinical trial planned
	Macopharma Theraflex ^®^	Methylene blue + visible light	- CE marked 2001
Red Blood Cells	Cerus Corporation INTERCEPT™	S303	- U.S. phase II and European phase III clinical trials completed
	Terumo BCT Mirasol ^®^	Riboflavin + UV light	- U.S. phase II clinical trial completed; phase III clinical trial planned

CE = Conformité Européenne

FDA = Food and Drug Administration

There is evidence for the PR systems available for platelets and plasma that most pathogens are inactivated, except for some non-enveloped viruses and certain bacterial strains ^18, 19^. Thus, there remains a need to screen donors for pathogens that may not be inactivated by the PR method. Additional critiques of PR technologies include a sizable cost increase compared to untreated products, negative effects on platelet function and count, acute respiratory distress syndrome (ARDS), and the potentially associated increased risk of bleeding in transfusion recipients ^17, 23^. The cost increase of PR technologies has been shown to be offset by the ability to extend the storage time of platelet products and the decreased rate of transfusion-related sepsis ^24^. While *in vitro* measures of adhesion and aggregation in PR platelets is comparable to untreated platelets, *in vivo* measures of post-transfusion corrected count increments and recovery are lower with PR platelets than untreated platelets ^25– 27^. Despite *in vitro* and *in vivo* study results, there remains equipoise over the clinical bleeding risk in patients who receive PR platelets compared to untreated platelets. For recipients of PR platelets, a Cochrane Review found no significant difference in clinically significant or severe bleeding, mortality, transfusion reactions, or adverse events (including sepsis and transfusion-transmitted infection) compared to recipients of untreated platelets ^27^. However, recipients of PR platelets generally require more platelet transfusions, thus more donor exposure, due to poor post-transfusion increments.

The numerous PR technologies available reflect the complexity of finding a balance between effective PR and preserving acceptable quality and functionality of the blood components. In addition to the decreased risk of bacterial contamination and TTIDs, PR technologies provide additional benefits of prevention of transfusion-associated graft-versus-host disease (GVHD), prevention of cytomegalovirus (CMV) disease transmission, and possible reduction of alloimmunization due to inactivation of white blood cells that remain in the blood products. While PR technologies may be a very good defense against emerging TTIDs, there remain concerns over cost, toxicity, alloimmunization, and cellular function (i.e. ** bleeding risk). As with most new technologies, additional studies are needed to ensure PR blood products are as effective as untreated products in preventing bleeding and adverse events in transfusion recipients. Current PR technologies are a step in the right direction; however, there remains a need to develop safer and better technologies that kill all pathogens.

## Bacterial contamination risk reduction

Transfusion-related microbial infections range in severity from a mild, transient temperature increase to acute lung injury, fulminant septic shock, and death. While there was only one fatality due to sepsis reported to the Food and Drug Administration (FDA) in fiscal year 2014, clinical sepsis is reported after 1 in every 100,000 platelet transfusions ^5, 16^. Blood collection centers have implemented a combination of techniques to reduce the risk of microbial contamination in the final blood product, including improved disinfection methods for the venipuncture site, introduction of the diversion pouch during the blood collection procedure, and automated bacterial culture of platelet products, as well as platelet additive solutions and PR systems. Newer developments in bacterial detection (i.e. nucleic acid testing and bio-chip technology) may be on the horizon but unfortunately have not been widely adopted or approved for use throughout the world. At the receiving end, hospital transfusion services have the ability to perform either culture or rapid, non-culture-based bacterial screening tests for platelet products that are at high risk for bacterial contamination at the time of issue. While the interventions described attempt to reduce the risk of fatal bacterial infection in Table 2, they do not provide protection from viral, protozoan, or other pathogens.

The leading sources of blood product contamination are skin bacteria from the venipuncture site during the blood collection procedure. Platelet products are especially susceptible to bacterial growth due to room temperature storage for up to 5 days. After disinfection with povidone iodine or isopropyl alcohol plus iodine tincture, only half of all donors will have remaining bacterial colonies when the venipuncture site is cultured ^1^. Unfortunately, bacteria in the deeper skin layers cannot be removed by simple skin disinfection, thus a diversion pouch is required in any collection system intended for preparation of a platelet product ^28^. The diversion pouch is integrally attached to the blood product collection set and collects the first few milliliters of whole blood, including any potential skin plug within the access needle, thus diverting the contaminating skin bacteria away from the final blood product container. The combination of skin disinfection with iodine-containing solutions and use of the diversion pouch effectively decreases the risk of bacterial contamination in platelet products ^29^.

Unfortunately, there remains a residual risk of bacterial contamination in platelet products despite the improved arm preparation techniques and use of the diversion pouch. Since 2004, the AABB Standards require testing of 100% of platelet products for bacterial contamination ^28^. For apheresis platelets, aliquots from the platelet product are generally drawn 18–24 hours after collection and used to inoculate aerobic cultures using one of the two bacterial detection systems cleared by the FDA for quality control testing of platelets. However, culture data and clinical reports show that even bacterial culture, the gold standard for bacterial detection, can fail to detect all contaminated platelet units ^30, 31^. For whole-blood-derived platelet products, an alternative to culture is the use of an FDA-cleared rapid bacterial detection test at the time of product issue. Unfortunately, these tests carry a high false-positive rate and result in the unnecessary discard of platelet products ^32^. However, the rapid bacterial detection tests have been successfully used to allow for the extension of apheresis platelet shelf life in times of medical necessity and help ensure that platelet products, especially day 4 and 5, are free of clinically significant levels of bacterial contamination ^33^. Thus, the Blood Product Advisory Committee (BPAC) has made recommendations to take precautions in order to decrease the risk of bacterial contamination of blood products.

In 2012, BPAC released a report recommending that blood centers and transfusion services either modify or perform additional testing on platelet products to help reduce the risk of recipient fatality due to bacterial contamination ^34^. Then, in 2014, the FDA released industry guidance in line with the BPAC report to enhance the safety of platelet transfusions ^35^. Recommendations for apheresis and pre-storage pooled platelets include testing for bacterial contamination using an FDA-cleared device no sooner than 24 hours post-collection and inoculating at least an aerobic culture medium, sampling the maximum volume permitted by the manufacturer of the culture device and considering increased sample volumes in proportion to collection volume (i.e. double and triple platelet collections) to maximize primary culture sensitivity, and adhering to the minimal incubation period described by the bacterial detection device manufacturer. Recommendations for whole-blood-derived platelets that are pooled at the time of issue and not previously tested for bacterial contamination include either culture as described for apheresis platelets or the use of a rapid bacterial detection device no sooner than 72 hours after collection. Thus, both the timing and the volume of the sample drawn for bacterial culture is important to help decrease the risk of sepsis due to bacterial contamination in platelet products.

While blood centers can adopt strategies to improve detection of bacterial contamination in platelet products, hospital transfusion services can take additional precautions to prevent septic transfusion reactions. As septic transfusion reactions and fatalities are more common on days 4 and 5 of storage, after initially low inoculums of bacteria are allowed to grow to clinically significant levels, the BPAC report and FDA guidance document also recommended the use of either a rapid bacterial screening test or re-culture of the platelet product on days 4 and 5 of storage ^34, 35^. Transfusion services have also successfully adopted a strategy of screening for bacterial contamination on day 4 or 5 of storage to extend the expiry of platelet products to 7 days when there is urgent clinical need with no increase in the rate of septic transfusion reactions ^33^. While no bacterial detection system is perfect, the combination of culture and rapid screening tests may provide the best risk reduction strategy for platelet transfusion-related septic reactions and safely extend the expiry date of the transfusion service’s most limited resource.

## TRALI risk mitigation strategies

TRALI is a severe transfusion reaction characterized by the acute onset of new, non-cardiogenic pulmonary edema that occurs within 6 hours of transfusion. Most cases of TRALI result from cognate antibodies between recipient human leukocyte antigens (HLA) on white blood cells and HLA antibodies in donor plasma. HLA antibodies are formed after exposure to foreign white blood cells. Thus, recipients of blood products replete with white blood cells, females who have been pregnant and exposed to fetal white blood cells, and recipients of solid organ or bone marrow transplants are most likely to develop HLA antibodies.

The prevalence of HLA antibodies in female donors is related to the number of prior pregnancies ^36^. Consequently, when comparing men and nulliparous women to previously pregnant donors, transfusion of blood components, especially apheresis plasma, carries a higher risk of inducing TRALI ^37^. While previous transfusion increases the overall risk of developing HLA antibodies, a study of donors who had received a transfusion greater than 12 months prior to enrollment revealed that the incidence of HLA antibodies was not significantly increased compared to non-transfused donors ^38^. Thus, a remote transfusion history in donors does not significantly contribute to an increased risk of HLA antibodies and these donors should not be excluded as part of TRALI risk mitigation strategies. Given the available evidence, in 2006, blood centers in the United States began restricting a large portion of plasma collections to males and nulliparous females. Subsequently, two separate analyses showed that a male-predominant plasma strategy has been associated with a reduction in TRALI cases ^39, 40^.

Then, in 2014, due to the observed residual risk of TRALI, the AABB Standard 5.4.1.2 stated that “plasma and whole blood for allogeneic transfusion shall be from males, females who have not been pregnant, or females who have been tested since their most recent pregnancy and results interpreted as negative for HLA antibodies” ^28^. Any donor who is found to be HLA antibody positive is not eligible for future donations of apheresis plasma or whole-blood-derived plasma for transfusion, while a negative result permits the release of all subsequent plasma components unless or until the donor becomes pregnant.

Therefore, blood collection facilities have two options to meet the AABB Standard: either perform HLA antibody screening on all female donors instead of taking a pregnancy history on all donors whose donation produces transfusable plasma components, or target HLA antibody testing for any female who has had any number of pregnancies carried to term or delivered. The estimated impact in loss of parous female donors must be weighed against targeted HLA testing to arrive at the best TRALI risk reduction strategy.

There has been some evidence suggesting human neutrophil antigen (HNA) antibodies may play a role in the development of TRALI, but due to their low prevalence in the donor population and the fact that they require specialized testing not conducive to large-scale screening, there is no compelling data to adopt screening for HNA antibodies ^41^. In addition, the fact that HLA antibody co-occurred in the majority of HNA antibody-positive donors suggests HNA-positive blood products may already be reduced as a consequence of HLA antibody screening.

Despite the current decreased incidence of TRALI with modified transfusion practices, it is still the leading cause of transfusion-related fatalities in the United States ^5^. However, it should be recognized that the FDA fatality reports through fiscal year 2014 include both TRALI and possible TRALI cases, the latter of which are most likely ARDS cases and not related to transfusion ^42^. Thus, plasma mitigation strategies will not decrease the incidence of possible TRALI. It is therefore paramount that clinicians recognize, diagnose, and report TRALI and TRALI-related mortality to the blood bank so that incidence, pathophysiology, and strategies to prevent this leading cause of transfusion-associated mortality can be further studied.

## Conclusion

The blood supply is the safest it has been in decades, yet blood centers and transfusion services continue to pursue massive efforts to prevent the infectious and non-infectious complications associated with blood product transfusion. One major challenge is to identify and stay one step ahead of emerging pathogens that threaten the safety of transfusable blood components. While blood centers continue to harness PR technologies and improve upon current pathogen detection techniques to enhance the safety of blood products, a challenge will be to keep the cost-benefit ratio of new technologies in check. Meanwhile, transfusion services will continue to struggle with the many non-infectious complications of transfusion. For example, despite the risk mitigation strategies implemented to date for plasma and platelet products, TRALI remains the leading cause of transfusion-related mortality, with red blood cell units now emerging as the implicated blood product. A challenge for the transfusion medicine community will be to further decrease the risk of TRALI while maintaining a healthy balance between the eligible donor pool and blood product inventory. While challenges remain for both blood centers and hospital transfusion services, the recent successes and strides made towards increasing the safety of the blood supply are noteworthy.

## References

[1] Fung MK: Technical Manual. 18th ed: AABB;2014.

[2] Roth WK Busch MP Schuller A: International survey on NAT testing of blood donations: expanding implementation and yield from 1999 to 2009. *Vox Sang.* 2012;102(1):82–90. 10.1111/j.1423-0410.2011.01506.x 21933190

[3] Zou S Stramer SL Dodd RY: Donor testing and risk: current prevalence, incidence, and residual risk of transfusion-transmissible agents in US allogeneic donations. *Transfus Med Rev.* 2012;26(2):119–28. 10.1016/j.tmrv.2011.07.007 21871776

[4] Kleinman S Stassinopoulos A: Risks associated with red blood cell transfusions: potential benefits from application of pathogen inactivation. *Transfusion.* 2015. 10.1111/trf.13259 26303806 PMC7169855

[5] Fatalities Reported to FDA Following Blood Collection and Transfusion: Annual Summary for Fiscal Year 2014.2014 Reference Source

[6] Goubran HA Burnouf T Stakiw J: Platelet microparticle: a sensitive physiological "fine tuning" balancing factor in health and disease. *Transfus Apher Sci.* 2015;52(1):12–8. 10.1016/j.transci.2014.12.015 25599988

[7] Varon D Shai E: Role of platelet-derived microparticles in angiogenesis and tumor progression. *Discov Med.* 2009;8(43):237–41. 20040277

[8] Rubin O Delobel J Prudent M: Red blood cell-derived microparticles isolated from blood units initiate and propagate thrombin generation. *Transfusion.* 2013;53(8):1744–54. 10.1111/trf.12008 23228139

[9] Rohde JM Dimcheff DE Blumberg N: Health care-associated infection after red blood cell transfusion: a systematic review and meta-analysis. *JAMA.* 2014;311(13):1317–26. 10.1001/jama.2014.2726 24691607 PMC4289152

[10] Lannan KL Sahler J Spinelli SL: Transfusion immunomodulation--the case for leukoreduced and (perhaps) washed transfusions. *Blood Cells Mol Dis.* 2013;50(1):61–8. 10.1016/j.bcmd.2012.08.009 22981700 PMC3508165

[11] Cholette JM Henrichs KF Alfieris GM: Washing red blood cells and platelets transfused in cardiac surgery reduces postoperative inflammation and number of transfusions: results of a prospective, randomized, controlled clinical trial. *Pediatr Crit Care Med.* 2012;13(3):290–9. 10.1097/PCC.0b013e31822f1586 21926663 PMC3839819

[12] Salpeter SR Buckley JS Chatterjee S: Impact of more restrictive blood transfusion strategies on clinical outcomes: a meta-analysis and systematic review. *Am J Med.* 2014;127(2):124–131.e3. 10.1016/j.amjmed.2013.09.017 24331453

[13] Carson JL Brooks MM Abbott JD: Liberal versus restrictive transfusion thresholds for patients with symptomatic coronary artery disease. *Am Heart J.* 2013;165(6):964-971.e1. 10.1016/j.ahj.2013.03.001 23708168 PMC3664840

[14] Lacroix J Hébert PC Fergusson DA: Age of transfused blood in critically ill adults. *N Engl J Med.* 2015;372(15):1410–8. 10.1056/NEJMoa1500704 25853745

[15] Steiner ME Ness PM Assmann SF: Effects of red-cell storage duration on patients undergoing cardiac surgery. *N Engl J Med.* 2015;372(15):1419–29. 10.1056/NEJMoa1414219 25853746 PMC5442442

[16] Snyder EL Stramer SL Benjamin RJ: The safety of the blood supply--time to raise the bar. *N Engl J Med.* 2015;372(20):1882–5. 10.1056/NEJMp1500154 25902384

[17] Jacobs MR Lazarus HM Maitta RW: The Safety of the Blood Supply--Time to Raise the Bar. *N Engl J Med.* 2015;373(9):882. 10.1056/NEJMc1507761 26308699

[18] Schmidt M Hourfar MK Sireis W: Evaluation of the effectiveness of a pathogen inactivation technology against clinically relevant transfusion-transmitted bacterial strains. *Transfusion.* 2015;55(9):2104–12. 10.1111/trf.13171 26013691

[19] Prowse CV: Component pathogen inactivation: a critical review. *Vox Sang.* 2013;104(3):183–99. 10.1111/j.1423-0410.2012.01662.x 23134556

[20] AuBuchon JP Prowse CV, AABB: Pathogen inactivation: the penultimate paradigm shift. Bethesda, Md.: AABB Press;2010;294 Reference Source

[21] Bolton-Maggs P (Ed) Poles D Thomas D: On behalf of the Serious Hazards of Transfusion (SHOT) Steering Group: The 2014 Annual SHOT Report.2015 Reference Source

[22] Bost V Chavarin P Boussoulade F: Independent evaluation of tolerance of therapeutic plasma inactivated by amotosalen-HCl-UVA (Intercept™) over a 5-year period of extensive delivery. *Vox Sang.* 2015;109(4):414–6. 10.1111/vox.12300 26031441

[23] Lozano M Cid J: Analysis of reasons for not implementing pathogen inactivation for platelet concentrates. *Transfus Clin Biol.* 2013;20(2):158–64. 10.1016/j.tracli.2013.02.017 23587612

[24] Girona-Llobera E Jimenez-Marco T Galmes-Trueba A: Reducing the financial impact of pathogen inactivation technology for platelet components: our experience. *Transfusion.* 2014;54(1):158–68. 10.1111/trf.12232 23656485

[25] Snyder E Raife T Lin L: Recovery and life span of ^111^indium-radiolabeled platelets treated with pathogen inactivation with amotosalen HCl (S-59) and ultraviolet A light. *Transfusion.* 2004;44(12):1732–40. 10.1111/j.0041-1132.2004.04145.x 15584988

[26] Slichter SJ Raife TJ Davis K: Platelets photochemically treated with amotosalen HCl and ultraviolet A light correct prolonged bleeding times in patients with thrombocytopenia. *Transfusion.* 2006;46(5):731–40. 10.1111/j.1537-2995.2006.00791.x 16686840

[27] Butler C Doree C Estcourt LJ: Pathogen-reduced platelets for the prevention of bleeding. *Cochrane Database Syst Rev.* 2013;3: CD009072. 10.1002/14651858.CD009072.pub2 23543569

[28] Standards for blood banks and transfusion services. 29th ed. Bethesda, MD: AABB;2014 Reference Source

[29] de Korte D Curvers J de Kort WL: Effects of skin disinfection method, deviation bag, and bacterial screening on clinical safety of platelet transfusions in the Netherlands. *Transfusion.* 2006;46(3):476–85. 10.1111/j.1537-2995.2006.00746.x 16533293

[30] Dumont LJ Kleinman S Murphy JR: Screening of single-donor apheresis platelets for bacterial contamination: the PASSPORT study results. *Transfusion.* 2010;50(3):589–99. 10.1111/j.1537-2995.2009.02460.x 19929862

[31] Jacobs MR Smith D Heaton WA: Detection of bacterial contamination in prestorage culture-negative apheresis platelets on day of issue with the Pan Genera Detection test. *Transfusion.* 2011;51(12):2573–82. 10.1111/j.1537-2995.2011.03308.x 21883265

[32] Harm SK Delaney M Charapata M: Routine use of a rapid test to detect bacteria at the time of issue for nonleukoreduced, whole blood-derived platelets. *Transfusion.* 2013;53(4):843–50. 10.1111/j.1537-2995.2012.03818.x 22845719

[33] Dunbar NM Kreuter JD Marx-Wood CR: Routine bacterial screening of apheresis platelets on Day 4 using a rapid test: a 4-year single-center experience. *Transfusion.* 2013;53(10):2307–13. 10.1111/trf.12083 23347110

[34] (BPAC) BPAC: Considerations for Options to Further Reduce the Risk of Bacterial Contamination in Platelets.2012 Reference Source

[35] U.S. Department of Health and Human Services FDA: Bacterial Detection Testing by Blood Collection Establishments and Transfusion Services to Enhance the Safety and Availability of Platelets for Transfusion.2014 Reference Source

[36] Triulzi DJ Kleinman S Kakaiya RM: The effect of previous pregnancy and transfusion on HLA alloimmunization in blood donors: implications for a transfusion-related acute lung injury risk reduction strategy. *Transfusion.* 2009;49(9):1825–35. 10.1111/j.1537-2995.2009.02206.x 19453983 PMC2841001

[37] Kleinman SH Triulzi DJ Murphy EL: The Leukocyte Antibody Prevalence Study-II (LAPS-II): a retrospective cohort study of transfusion-related acute lung injury in recipients of high-plasma-volume human leukocyte antigen antibody-positive or -negative components. *Transfusion.* 2011;51(10):2078–91. 10.1111/j.1537-2995.2011.03120.x 21446938 PMC3606005

[38] Kakaiya RM Triulzi DJ Wright DJ: Prevalence of HLA antibodies in remotely transfused or alloexposed volunteer blood donors. *Transfusion.* 2010;50(6):1328–34. 10.1111/j.1537-2995.2009.02556.x 20070615 PMC2891258

[39] Schmickl CN Mastrobuoni S Filippidis FT: Male-predominant plasma transfusion strategy for preventing transfusion-related acute lung injury: a systematic review. *Crit Care Med.* 2015;43(1):205–25. 10.1097/CCM.0000000000000675 25514705

[40] Müller MC van Stein D Binnekade JM: Low-risk transfusion-related acute lung injury donor strategies and the impact on the onset of transfusion-related acute lung injury: a meta-analysis. *Transfusion.* 2015;55(1):164–75. 10.1111/trf.12816 25135630

[41] Gottschall JL Triulzi DJ Curtis B: The frequency and specificity of human neutrophil antigen antibodies in a blood donor population. *Transfusion.* 2011;51(4):820–7. 10.1111/j.1537-2995.2010.02913.x 20977484 PMC3583295

[42] Toy P Bacchetti P Grimes B: Recipient clinical risk factors predominate in possible transfusion-related acute lung injury. *Transfusion.* 2015;55(5):947–52. 10.1111/trf.12954 25488517 PMC4428945

