# Supplementary material for: Recent Advances in Preventing Adverse Reactions to Transfusion
Source: F1000Res. 2015 Dec 17;4:F1000 Faculty Rev-1469. [Version 1] doi: 10.12688/f1000research.7048.1 (PMC4815612; doi:10.12688/f1000research.7048.1)
Supplement: Peer review history

# Peer review: Referee response for version 1

**DOI:** 10.5256/f1000research.7588.r11483

**Published:** 2015-12-17

**Authors:** Neil Blumberg

**Affiliations:** 1 Department of Pathology and Laboratory Medicine, University of Rochester Medical Center, Rochester, New York, 14642, USA

The relatively recently described complications of nosocomial infection, thrombosis and multi-organ failure that are caused, in part, by allogeneic red cell transfusion affect 1-2% of transfused patients, at minimum (1-4). These are severe and life threatening acute morbidities that are rarely mentioned in current textbooks and reviews, but well characterized in randomized trials of restrictive transfusion practices (2,3), autologous transfusion (5) and leukoreduced (1) transfusions. Thus mitigating the side effects of transfusion includes strategies such as restrictive red cell transfusion where validated in randomized trials, universal leukoreduction of transfusions, and implementation of blood management programs including techniques for minimizing the need for allogeneic transfusion. Such techniques can include various types of autologous transfusion (normovolemic hemodilution, cell salvage) and minimization of iatrogenic or surgical blood loss, as well as anemia management and pharmacologic approaches that mitigate anemia and thrombocytopenia.  

There is also preliminary data that removal of supernatant from transfused red cells and platelets may benefit patients undergoing cardiac surgery (6) and those treated for acute leukemia (7). Animal models support these possible benefits of washed transfusions (8).

In addition, there is a growing body of evidence that ABO non-identical transfusions interfere with normal hemostasis, increase bleeding and mortality in hospitalized patients (9-13). Use of ABO identical transfusions is associated with reduced transfusion reactions, lung injury, alloimmunization and death in epidemiologic observational studies. While these data are still preliminary, they derive from multiple institutions and clinical settings, and have credible mechanistic explanations. ABO identical transfusions may be one key strategy to improve clinical outcomes in the future. (14)

I have read this submission. I believe that I have an appropriate level of expertise to confirm that it is of an acceptable scientific standard.

## References

[1] FergussonDKhannaMPTinmouthAHébertPC: Transfusion of leukoreduced red blood cells may decrease postoperative infections: two meta-analyses of randomized controlled trials. *Can J Anaesth*.2004;51(5) : 10.1007/BF03018302 417-24 10.1007/BF03018302 15128625

[2] SalpeterSRBuckleyJSChatterjeeS: Impact of more restrictive blood transfusion strategies on clinical outcomes: a meta-analysis and systematic review. *Am J Med*.2014;127(2) : 10.1016/j.amjmed.2013.09.017 124-131.e3 10.1016/j.amjmed.2013.09.017 24331453

[3] RohdeJMDimcheffDEBlumbergNSaintSLangaKMKuhnLHicknerARogersMA: Health care-associated infection after red blood cell transfusion: a systematic review and meta-analysis. *JAMA*.2014;311(13) : 10.1001/jama.2014.2726 1317-26 10.1001/jama.2014.2726 24691607PMC4289152

[4] RobertsonCSHannayHJYamalJMGopinathSGoodmanJCTilleyBCBaldwinARivera LaraLSaucedo-CrespoHAhmedOSadasivanSPonceLCruz-NavarroJShahinHAisikuIPDoshiPValadkaANeipertLWaguspackJMRubinMLBenoitJSSwankP: Effect of erythropoietin and transfusion threshold on neurological recovery after traumatic brain injury: a randomized clinical trial. *JAMA*.2014;312(1) : 10.1001/jama.2014.6490 36-47 10.1001/jama.2014.6490 25058216PMC4113910

[5] VanderlindeESHealJMBlumbergN: Autologous transfusion. *BMJ*.2002;324(7340) :772-5 1192316210.1136/bmj.324.7340.772PMC1122708

[6] CholetteJMHenrichsKFAlfierisGMPowersKSPhippsRSpinelliSLSwartzMGensiniFDaughertyLENazarianERubensteinJSSweeneyDEatonMLernerNBBlumbergN: Washing red blood cells and platelets transfused in cardiac surgery reduces postoperative inflammation and number of transfusions: results of a prospective, randomized, controlled clinical trial. *Pediatr Crit Care Med*.2012;13(3) : 10.1097/PCC.0b013e31822f173c 290-9 10.1097/PCC.0b013e31822f173c 21926663PMC3839819

[7] BlumbergNHealJMRoweJM: A randomized trial of washed red blood cell and platelet transfusions in adult acute leukemia [ISRCTN76536440]. *BMC Blood Disord*.2004;4(1) : 10.1186/1471-2326-4-6 6 10.1186/1471-2326-4-6 15588315PMC544564

[8] Cortés-PuchIWangDSunJSolomonSBRemyKEFernandezMFengJKaniasTBellaviaLSincharDPerlegasASolomonMAKelleyWEPopovskyMAGladwinMTKim-ShapiroDBKleinHGNatansonC: Washing older blood units before transfusion reduces plasma iron and improves outcomes in experimental canine pneumonia. *Blood*.2014;123(9) : 10.1182/blood-2013-11-539353 1403-11 10.1182/blood-2013-11-539353 24366359PMC3938151

[9] BlumbergNHealJMHicksGLRisherWH: Association of ABO-mismatched platelet transfusions with morbidity and mortality in cardiac surgery. *Transfusion*.2001;41(6) :790-3 1139982110.1046/j.1537-2995.2001.41060790.x

[10] InabaKBrancoBCRheePHolcombJBBlackbourneLHShulmanINelsonJDemetriadesD: Impact of ABO-identical vs ABO-compatible nonidentical plasma transfusion in trauma patients. *Arch Surg*.2010;145(9) : 10.1001/archsurg.2010.175 899-906 10.1001/archsurg.2010.175 20855762

[11] ShanwellAAnderssonTMRostgaardKEdgrenGHjalgrimHNordaRMelbyeMNyrénOReillyM: Post-transfusion mortality among recipients of ABO-compatible but non-identical plasma. *Vox Sang*.2009;96(4) : 10.1111/j.1423-0410.2009.01167.x 316-23 10.1111/j.1423-0410.2009.01167.x 19254234

[12] HenrichsKFHowkNMaselDSThayerMRefaaiMAKirkleySAHealJMBlumbergN: Providing ABO-identical platelets and cryoprecipitate to (almost) all patients: approach, logistics, and associated decreases in transfusion reaction and red blood cell alloimmunization incidence. *Transfusion*.2012;52(3) : 10.1111/j.1537-2995.2011.03329.x 635-40 10.1111/j.1537-2995.2011.03329.x 21895675PMC3235249

[13] ZaffutoBJConleyGWConnollyGCHenrichsKFFrancisCWHealJMBlumbergNRefaaiMA: ABO-immune complex formation and impact on platelet function, red cell structural integrity and haemostasis: an in vitro model of ABO non-identical transfusion. *Vox Sang*.2015; 10.1111/vox.12354 10.1111/vox.12354 26528766

[14] PaiMCookRBartyREikelboomJLeeKAHeddleN: Exposure to ABO-nonidentical blood associated with increased in-hospital mortality in patients with group A blood. *Transfusion*.2015; 10.1111/trf.13376 10.1111/trf.13376 26472598

# Peer review: Referee response for version 1

**DOI:** 10.5256/f1000research.7588.r11482

**Published:** 2015-12-17

**Authors:** Jerard Seghatchian

**Affiliations:** 1 Audit/Inspection and DDR Strategy, International Consultancy in Blood Components Quality/Safety, London, UK

I have read this submission. I believe that I have an appropriate level of expertise to confirm that it is of an acceptable scientific standard.

# Peer review: Referee response for version 1

**DOI:** 10.5256/f1000research.7588.r11480

**Published:** 2015-12-17

**Authors:** Pearl Toy

**Affiliations:** 1 Department of Laboratory Medicine, University of California, San Francisco, San Francisco, California, 94143, USA

I have read this submission. I believe that I have an appropriate level of expertise to confirm that it is of an acceptable scientific standard.

# Peer review: Referee response for version 1

**DOI:** 10.5256/f1000research.7588.r11481

**Published:** 2015-12-17

**Authors:** Erhard Seifried

**Affiliations:** 1 German Red Cross Blood Transfusion Services Baden-Wuerttemberg, Frankfurt, D-60528, Germany

I have read this submission. I believe that I have an appropriate level of expertise to confirm that it is of an acceptable scientific standard.
